# Pomegranate (*Punica granatum* L.) and Its Rich Ellagitannins as Potential Inhibitors in Ulcerative Colitis

**DOI:** 10.3390/ijms242417538

**Published:** 2023-12-16

**Authors:** Huimin Li, Jingya Ruan, Jiayan Huang, Dingshan Yang, Haiyang Yu, Yuzheng Wu, Yi Zhang, Tao Wang

**Affiliations:** 1 State Key Laboratory of Component-Based Chinese Medicine, Tianjin University of Traditional Chinese Medicine, 10 Poyanghu Road, West Area, Tuanbo New Town, Jinghai District, Tianjin 301617, China; 15380711687@163.com (H.L.); ruanjingya@tjutcm.edu.cn (J.R.); huangjiayan1127@163.com (J.H.); hyyu@tjutcm.edu.cn (H.Y.); wyz2019@tjutcm.edu.cn (Y.W.); 2 Tianjin Key Laboratory of TCM Chemistry and Analysis, Tianjin University of Traditional Chinese Medicine, 10 Poyanghu Road, West Area, Tuanbo New Town, Jinghai District, Tianjin 301617, China; yangdingshan1996@163.com

**Keywords:** ulcerative colitis, pomegranate extract, ellagitannins, punicalagin, ellagic acid, research progress

## Abstract

Ulcerative colitis, an immune-mediated inflammatory disease of the gastrointestinal tract, places a significant financial burden on patients and the healthcare system. Recently, reviews of the pomegranate and the abundant medicinal applications of its ellagitannins, as well as its pharmacological action, phytochemicals, metabolism, and pharmacokinetics, have been completed. However, summaries on their anti-ulcerative colitis effects are lacking. Numerous preclinical animal investigations and clinical human trial reports demonstrated the specific therapeutic effects of pomegranate and the effect of its ellagitannins against ulcerative colitis. According to the literature collected by Sci-finder and PubMed databases over the past 20 years, this is the first review that has compiled references regarding how the rich ellagitannins found in pomegranate have altered the ulcerative colitis. It was suggested that the various parts of pomegranates and their rich ellagitannins (especially their primary components, punicalagin, and ellagic acid) can inhibit oxidant and inflammatory processes, regulate the intestinal barrier and flora, and provide an anti-ulcerative colitis resource through dietary management.

## 1. Introduction

Ulcerative colitis (UC) is a chronic inflammatory bowel disease (IBD) caused by genetics, gut microbiota, the environment, and other factors. Adults between the ages of 30 and 40 years old are prone to the disease. It is difficult to cure, as it often lasts during an entire lifetime [1]. UC lesions are usually confined to the mucosal layer, causing superficial damage to the intestinal wall. It begins in the rectum and progresses toward the proximal colon, which is clinically characterized by bloody diarrhea [2]. Epidemiological studies showed that the incidence rate of UC increased year by year, and it has been listed as a “modern refractory disease” by the World Health Organization [3]. At present, 5-aminosalicylic acid, glucocorticoid and immunosuppressive drugs, and colon resection are mainly used to treat UC [4]. Each of these treatment options has drawbacks, including the development of drug resistance, patients’ intolerance of postoperative storage bag anastomotic leaking, stomach infections, intestinal obstructions, and other problems [5]. According to estimates, the lifetime treatment costs of UC patients are comparable to those of patients with cancer and cardiovascular disease, which has a significant influence on patients’ quality of life and poses significant issues for public health. As a result, the focus of current UC research is centered around finding long-term effective anti-UC treatment medications with minimal medical side effects and medical burden. Recently, scientists have been more closely observing the development of biologically active compounds abundant in diet or plants as potential medications for the prevention and treatment of UC.

Tannin, also known as tannic acid, is a kind of water-soluble polyphenolic compound with a relative molecular weight of 500–3000 Da. It can be divided into hydrolyzed tannins and condensed tannins. Currently, it is widely believed that hydrolyzed tannins are the main bioactive types. By precipitation interaction with tissue proteins, the hydroxyl group, or other functional groups of tannins, can produce a protective coating of the tannin–protein complex locally, serving to resist both mechanical and chemical damage as well as encourage mucosal regeneration, thereby protecting gastrointestinal tissue [6]. In 2020, González–Quilen reviewed the protective effects and related mechanisms of condensed tannins in IBD and found that they could alleviate IBD symptoms by inhibiting inflammation and protecting the intestinal barrier [7]. However, to date, no relevant induction and summary of hydrolyzed tannins on UC have been reported.

Hydrolyzed tannins are widely distributed in tea, nuts, grains [8], pomegranate, strawberries, raspberries, and other foods [9]. The core structure of hydrolyzed tannins is comprised of polyols, such as glucose. According to whether the hydroxyl of polyols is replaced by gallic acid or esterified by hexahydroxydiphenyl acid, these components are divided into gallic tannins and ellagitannins [10]. Among them, the latter are more common. They are anti-inflammatory and antibacterial, and they possess antioxidants and anticancer effects, as well as various other pharmacological effects [11]. Some ellagitannins have been used in the clinic in the meantime. For instance, as the main component of the Mongolian drug “Zhachong Thirteen Taste Pills,” ellagic acid (EA) has been clinically utilized to treat ischemic cerebrovascular illnesses [12].

**Figure 1:**
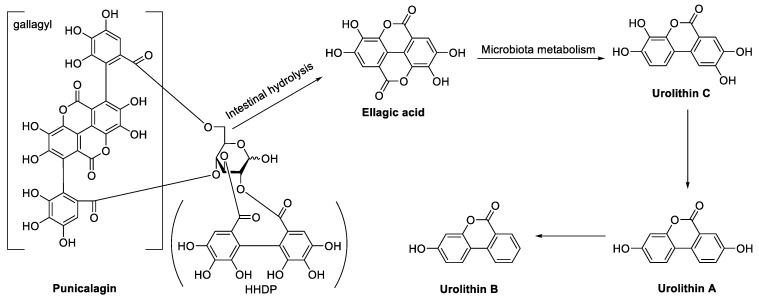
The hydrolytic metabolic pathway of ellagitannin in the intestine was explained by using punicalagin as an example.

Pomegranate is an edible and medicinal dual-use plant with important therapeutic benefits in daily health care and disease prevention [13,14,15]. Its different parts (fruit, flower, peel) are all rich in ellagitannins such as punicalagin and EA (Figure 1), as well as antioxidants and other active ingredients that cause its multiple biological effects, including antidiarrhea and antiinflammation, as well as acute and chronic intestinal inflammation intervention [16,17,18]. Even though their potential anti-UC activity has been discovered, summaries of relevant mechanisms are still lacking. In addition, ellagitannins usually exist in polymer form, accompanied by poor solubility [19], inevitably affecting their bioavailability and pharmacological activity in vivo. Therefore, research on their metabolites has also become one of the hot topics in recent years.

This study summarized the in vivo metabolic process, the UC-relieving properties, and the action mechanism of pomegranate, along with its rich ellagitannins.

## 2. In Vivo Metabolic Process of Ellagitannins in Pomegranate

As one kind of hydrolyzed tannin, ellagitannins are unstable in the pH environment of the gastrointestinal tract and partially hydrolyzed to EA. After oral administration, ellagitannins are first transformed to EA in the stomach and proximal small intestine partially. EA has a low solubility and can only be partially absorbed into the blood. Unabsorbed EA and ellagitannins are then processed by the gut bacteria to create Urolithins A, B, C, etc. (Figure 1). Compared to ellagitannins, urolithins are better absorbed in the digestive tract and can enter the systemic circulation to have a biological effect [20].

Urolithins can be used as a biomarker for ellagitannin intake. According to the different types of urolithin produced by individual metabolism, the metabolisms of ellagitannins can be divided into three types: Type A (producing Urolithin A and its derivatives), Type B (producing Urolithins A and B, as well as Isourolithin A and its derivatives), and Type 0 (not producing) [21]. According to the research conducted by Seeram [22], 18 healthy volunteers were provided 180 mL of pomegranate juice (PJ) concentrate, and urine samples were collected and analyzed on the same day. Among them, 11 were observed to possess Type A metabolism as Urolithin A and its derivatives were detected in the urine samples. Urolithins A and B, as well as their derivatives, were detected in three volunteers, which indicated that they possessed Type B metabolism. The others are suggested to possess Type 0 metabolism. According to the genus-level correlation analysis between the metabolism type and the content of urolithins and the gut microbiota [23,24], the diversity and richness of gut microbiota for Type 0 metabolism were less than those of Type A and Type B. *Gordonibacter* was substantially more prevalent in Type A and Type B than it was in Type 0. In Type 0, the *Akkermansia* was absent. A total of 20 genera were positively associated with Isourolithin A and Urolithin B, while 27 genera were significantly positively correlated with Urolithin A, according to the genus-level correlation analysis between the type and content of urolithins and the gut microbiota.

How do ellagitannins exert biological effects on people with Type 0 metabolism? It has been found that in the gastrointestinal tract, the hydrolysis rate of ester bonds in ellagitannins is relatively slow, which extends their retention time in the gastrointestinal tract, thus exerting a local role [25]. According to an in vivo investigation by Seeram [26], after volunteers drank 180 mL of PJ containing 25 mg of EA for 1 h, the highest concentration of EA in human plasma was only 31.9 ng/mL, and it vanished after 4 h. The above results suggested that the efficacy of ellagitannins did not depend mainly on their blood concentration. Therefore, low bioavailability of this kind of compound may be an important problem for patients with systemic inflammatory diseases. Even in circumstances of low bioavailability of substances, a local concentration of compounds in the intestinal cavity may be sufficient for UC patients—even those with Type 0 metabolism—because UC is restricted to the intestinal mucosa [27]. This may be due to the interaction between the hydroxyl groups in the ellagitannin structure and the proteins of the intestinal mucosa, which results in the formation of a protective film or the partial absorption of tannic acid into the bloodstream to exert biological activity in the intestine.

To summarize, ellagitannins are partially hydrolyzed in the body, releasing free EA, and part of them can be directly absorbed into the circulation to play a role. Ellagitannins, or EAs that have not been hydrolyzed, could locally form a protective film of an ellagitannin–protein complex with intestinal epithelial cells through a covalent bond, hydrogen bond, and hydrophobic action, helping to prevent mucosal regeneration from being damaged by mechanical or chemical stimulation, thus protecting gastrointestinal tissue [6]. The leftover ellagitannins and EA are further degraded by the gut microbiota of the intestine to create urolithin, which can be absorbed into the blood and enter the systemic circulation to have a physiological effect. EA is rapidly eliminated in vivo, hence the dosage or frequency of its administration must be increased to maintain blood concentration.

## 3. Pharmacological Effect and Mechanism of Pomegranate and Its Ellagitannins on UC

In this article, the anti-UC effects and mechanisms of several pomegranate parts and the fruit’s abundant ellagitannins have been reviewed from the perspectives of its antioxidants, its anti-inflammatory properties, and its ability to enhance the intestinal barrier and regulate gut microbiota (Table 1).

### 3.1. Antioxidant Effects and Mechanisms

Immune cells such as neutrophils and macrophages would become active in the intestinal cavity upon invasion by microorganisms and pathogens. The active immune cells would then lead to the production of reactive oxygen species (ROS), superoxide anion-free radicals (O2•−), hydroxyl-free radicals (•OH), and other reactive oxygen species (ROM) metabolites [44]. The booming ROM would further cause the oxidation of lipids, proteins, and DNA, as well as damage to the structure and function of cells and the colon tissues. As a result, the occurrence and development of inflammatory diseases were promoted [45]. According to studies, UC patients with colon tissue inflammation create more ROM compared to healthy people, which has been repeatedly confirmed in UC animal models. It was suggested that the use of antioxidants may have potential therapeutic effects for UC [46].

**Figure 2:**
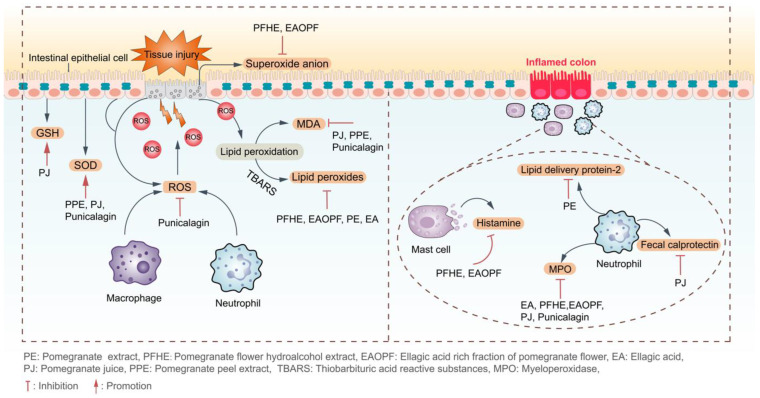
Pomegranate and its rich ellagitannins displayed anti-UC effects by exerting antioxidant effects and inhibiting the infiltration of different immune cells.

Ellagitannins, which scavenge free radicals, prevent lipid peroxidation, and activate antioxidant enzyme activity, can inhibit the overproduction of ROM and contribute to the improvement of UC (Figure 2).

#### 3.1.1. Clearing Free Radicals Directly

Free radicals are effectively scavenged by tannins from food or plants. Tannins’ hydroxyl group can provide proton binding to excess free radicals, stopping their chain reaction and causing a substantial antioxidant effect [47].

In vitro, the antioxidant activity of tannin-rich substances can be studied by chemical methods such as 2,2′-diazobenzothiazolin-bi-3-ethyl benzothiazole line-6-sulfonic acid-free radical method (ABTS), ferric ion-reducing antioxidant power (FRAP), and the 1,1-diphenyl-2-trinitrophenylhydrazine free radical method (DPPH), with the free radical clearance rate as an indicator [48]. A high-performance liquid chromatography (HPLC) was used and combined with ABTS, DPPH, and FRAP methods to evaluate the antioxidant activity of PJ [28]. They discovered that hydrolyzed tannin is the main antioxidant component in PJ. Punicalagin possessed strong antioxidant activity in vitro.

However, in vitro antioxidant research cannot comprehensively evaluate the antioxidant effect of ellagitannins. The main reason is that their bioavailability is influenced by complex physiological processes such as absorption, distribution, and metabolism in the body, while in vitro chemical methods cannot exclude the interference of these factors. Therefore, analyzing the antioxidant activity of ellagitannins at the animal level is more convincing. The Elisa method was conducted to detect the secretion of ROS in the colon tissue of DSS-induced mice, and the results showed that ellagitannins could inhibit the rise in ROS levels [29].

The nitrochlorotetrazolium blue (NBT) method was used to measure the content of superoxide anion in the colon tissue of mice [17]. The findings suggested that the anti-UC actions of pomegranate flower hydroalcohol extract (PFHE) and ellagic acid-rich fraction of pomegranate flower (EAOPF) could significantly reduce the increase of superoxide anion levels by DSS simulation, playing an antioxidant role.

#### 3.1.2. Inhibiting Lipid Peroxidation

Malondialdehyde (MDA), 4-hydroxynonenal (4-HNE), and other lipid peroxides can be produced when excess ROM reacts with polyunsaturated fatty acids on the cell membrane [49].

In the lipid peroxidation reaction, polyphenols can neutralize lipid peroxide-free radicals and alkoxy-free radicals and lower the amount of lipid peroxides produced [50]. The level of peroxides in colon tissue was measured by the thiobarbituric acid reactive substances (TBARS) method. It was found that compared with the normal group, the level of TBARS in colon tissue of model group mice induced by dextran sulfate sodium salt (DSS) significantly increased, while after the intervention of PFHE and EA-rich components, the level of TBARS significantly decreased [17]. As a result, the accumulation of lipid peroxides in the body was reduced. The level of lipid peroxidation in the colon tissue of rats was detected by the TBARS method. It was discovered that pomegranate extract (PE) dramatically reduced the level of TBARS in the colon tissue of rats compared with the model group [25]. Urolithin tended to decrease TBARS in the gut, although this trend was not statistically significant. It was found that EA could inhibit the increase of TBARS in the colon tissue of DSS-induced colitis rats [30]. It was also found that PJ and its main component, punicalagin, could reduce the level of MDA in the intestinal tissue of UC rats modeled by 2,4-dinitrobenzene sulfonic acid (DNBS), alleviating the intestinal mucosal damage caused by oxidative stress [31].

In a study by Al Subory, the experiment was divided into a normal group (mice were provided normal feed), a feed group containing 5% pomegranate peel extract (PPE), and a feed group containing 10% PPE. After 37 days, the mice were euthanized and relevant indicators were tested. The results demonstrated that compared to the control group, the amount of MDA in the mice’s plasma and colon tissue decreased in the feed group with 5% PPE and the feed group with 10% PPE, while the latter effect was more obvious [32]. Additionally, research also found that punicalagin prevented the rise in MDA levels in UC mice’s colon tissue caused by DSS modeling [29]. In conclusion, the above research results suggested that the daily intake of ellagitannins with antioxidant properties can prevent and treat UC.

#### 3.1.3. Activating the Activity of Antioxidant Enzyme

ROMs are also produced by cells through typical metabolic activities. Cells and tissues can release protective enzymes and non-enzymatic antioxidants to promptly break them down. During the inflammatory process, the production of ROM increases abnormally, causing tissue damage. The gastrointestinal tract is one of the key sources of ROM production. Mitochondria are the main site of ROM production in most mammalian cells. Isomers of superoxide dismutase (SOD1 and SOD2), glutathione peroxidase (GPX), and glutathione reductase (GSH), produced by colonic epithelial cells, are the first line of antioxidant defense for intestinal tissues against oxidative stress [51].

Pomegranate peel accounts for about 50% of the weight of pomegranate fruit and is an important source of ellagitannins. According to reports, the mouse diet containing 10% PPE can increase the expression of SOD1 and SOD2 in the intestinal tissue of UC mice. The findings suggested that a diet containing pomegranate peel may merit further investigation as a preventative measure for intestinal disorders caused by oxidative stress [32]. It was found that PJ and punicalagin can alleviate the reduction of SOD levels in colon tissue caused by DNBS modeling and play an antioxidant role [31]. GSH is the main intracellular antioxidant that directly neutralizes excess free radicals and ROM. In the rat UC model induced by 2,4,6-trinitrobenzene sulfonic acid (TNBS), PJ, as an antioxidant, can increase the content of GSH in the rat serum and alleviate the symptoms of UC [33]. According to Larrosa’s study [25], EA, hydrolyzed from ellagitannins in vivo showed strong antioxidant activity, and urolithin produced by its further metabolism had weaker antioxidant activity. Therefore, it was hypothesized that PE and its hydrolysate, EA, rather than urolithin, were the biological substances that decreased the amount of oxidative stress levels in DSS-induced rat plasma and colon tissue. In addition, the antioxidant activity of ellagitannins is directly related to the number of their hydroxylation. The presence of *o*-phenolic hydroxyl groups significantly improved its antioxidant activity, but the substitution of glycose will reduce its antioxidant effects [52].

In summary, pomegranate and its ellagitannins can exert antioxidant effects in a UC symptom by directly clearing free radicals, inhibiting peroxide production, and promoting the release of antioxidant enzymes. The hydroxyl groups are important functional groups for ellagitannins to exert antioxidant activity. The increase in hydroxyl number enhanced its ability to capture free radicals, resulting in a corresponding enhancement in antioxidant activity. The ortho–dihydroxy structure showed high stability and could significantly increase antioxidant activity.

### 3.2. Anti-Inflammatory Effects and Mechanisms

As the body’s natural physiological defense mechanism, the inflammatory response plays a key role in mediating the body’s defense against infections, tissue repair, and re-establishing homeostasis [53]. It is generally believed that a controllable inflammatory response is beneficial to the body, such as preventing infection and maintaining normal physiological functions. However, if inflammation cannot be effectively controlled, it can cause excessive tissue damage and chronic inflammation. It may even be fatal, causing septic shock [54]. According to reports, there is a persistent inflammatory response in the intestinal lesion site of UC patients, which is a key factor in the development of UC [55]. Based on its characteristics, a sustained treatment plan is needed. The daily consumption of ellagitannins is gaining popularity as a method to prevent and treat inflammatory illnesses. Its mode of action may involve controlling immunological response, controlling signaling pathways related to inflammation, and healing colorectal tissue damage brought on by an overactive inflammatory response.

#### 3.2.1. Regulating Immune Response

The gastrointestinal tract is the main site of interaction between the human immune system and microorganisms. In the colon, many types of immune cells release pro- and anti-inflammatory mediators, which are tightly regulated to control the ablation and dissemination of inflammation (Figure 2).

##### Inhibition of Neutrophil Infiltration

The characteristic of UC is inflammation of the colon mucosa, accompanied by inflammatory cell infiltration. Among them, neutrophils are first recruited to immune cells in the inflammatory region. Studies have detected the presence of neutrophils in the area of the intestinal mucosal injury area in UC patients [56]. Fecal calprotectin (FC) is a calcium-binding cytoplasmic protein mainly derived from neutrophils in the intestine. It is a marker of intestinal mucosal inflammation, and its expression level is positively correlated with the result of endoscopic and histological assays [57]. It was found that in clinically stable IBD patients, although their symptoms were relieved, the levels of inflammatory factors in the body were still high [34]. PJ that mainly contained ellagitannins was found to downregulate the FC level in IBD patients after 12 weeks of intervention as compared to the control group receiving a placebo. In addition, human lipid delivery protein-2 (LCN2) expressed in neutrophils is considered to be a highly sensitive biomarker for intestinal mucosal inflammation. After 8 weeks of administration of PE to IL-10 knockout mice, the levels of LCN2 in the feces and plasma of the mice were detected. The results showed that PE could downregulate the level of LCN2 in the feces and plasma of IL-10 knockout mice and bring model group mice’s LCN2 levels back to those of the control group after 8 weeks of administration [35].

Myeloperoxidase (MPO) is an oxidase mainly secreted by neutrophils and has been used as an indicator of neutrophil flow into intestinal inflammatory tissue [58]. It was suggested that PJ could reduce the increase of MPO levels in rat serum caused by TNBS modeling [33]. It was found that DNBS modeling caused the increase of MPO content in the intestinal tissue of UC rats [31], and PJ and punicalagin tended to reduce the level of MPO. The infiltration of neutrophils was indirectly reflected by measuring the content of MPO in colon tissue [36]. Compared to the DSS-induced acute UC model group, the group with 2% EA in the mouse meal tended to diminish the expression of MPO in colon tissue, but there was no statistically significant difference. A TNBS-induced rat chronic UC model (14 days) was established by Rosillo. It was found that the administration of 250 mg/kg and 500 mg/kg of PE, as well as the administration of 10 mg/kg of EA, could significantly reduce the production of MPO in the colon tissue of TNBS-induced rats [37]. Similarly, it was found that in the colon of DSS-induced acute UC model group mice (7 days), high- and low-dose groups (200 mg/kg, 100 mg/kg) of PFHE and the high- and low-dose groups (200 mg/kg, 100 mg/kg) of EAOPF both reduced the expression level of MPO in the colon in a dose-dependent manner compared with the normal group mice [17]. Moreover, compared with PFHE, the group rich in EA had a more significant effect on the reduction of MPO level, suggesting that EA may be the main active component of pomegranate flower to play an anti-UC effect.

In conclusion, we found that the anti-inflammatory effect of EA was unstable in the DSS-induced acute mouse UC model. The main reason may be that EA has poor water solubility and low permeability leading to its unstable absorption in vivo and low bioavailability. Additionally, EA was eliminated quickly in the body, resulting in a short stay time in the body, especially in the acute mouse UC model where the dosing period is usually 7 days. All of the above factors would result in a lack of EA in the colon tissue, reaching the effective concentration to exert its function. Moreover, individual differences between mice can also affect the experimental results. Therefore, the therapeutic concentration of ellagitannins and EA in the intestinal tissue needs to be adjusted and extended according to the actual situation to maintain a relatively stable concentration in the body, which may be of great benefit in the treatment of diseases like chronic UC.

##### Inhibition of Mast Cell Degranulation

Mast cell is an immune cell widely distributed in the gastrointestinal tract mainly existing in the mucosal lamina propria and submucosa of the gastrointestinal tract [59]. The number of mast cells in the intestinal mucosa of IBD patients was significantly higher than that of healthy people. More studies have shown that the mast cell was involved in the occurrence and development of IBD [60]. When activated, mast cells play a significant part in the inflammatory response process of UC by releasing various inflammatory compounds such as histamine, chemokines, etc. [61]. Histamine release from intestinal tissue is a specific marker of mast cell degranulation. It was found that after DSS was provided to mice, the histamine content in the colon tissue of mice was significantly increased [17]. The intervention with 200 mg/kg and 100 mg/kg of PFHE, as well as 200 mg/kg and 100 mg/kg of EAOPF, could reverse this situation. Furthermore, treatment with sodium cromoglycate, a mast cell stabilizer, also alleviated DSS-induced colonic injury. The above results indicated that PFHE and its EA-rich component may play an anti-UC role by stabilizing mast cells, preventing their degranulation and reducing the production of inflammatory mediators.

#### 3.2.2. Influencing on the Inflammatory Signaling Pathway

**Figure 3:**
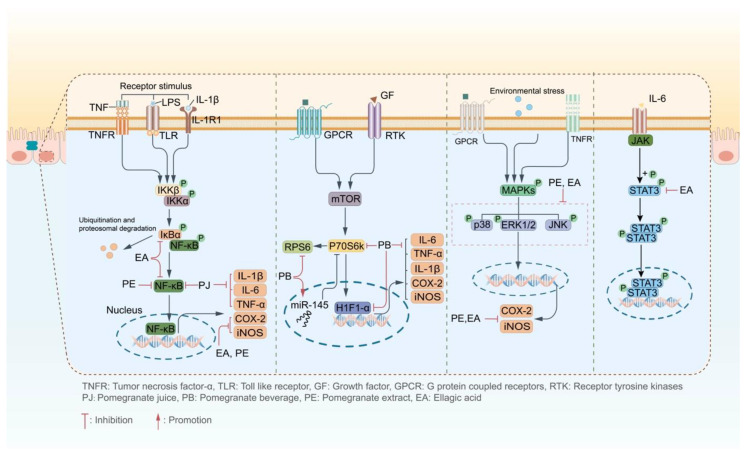
Pomegranate and its rich ellagitannins exerted anti-UC effects by inhibiting different inflammatory-signaling pathways (P: phosphorylated).

In recent years, many experiments have shown that ellagitannins could alleviate intestinal inflammation by inhibiting inflammatory-signaling pathway-related proteins, such as nuclear transcription factors κB (NF-κB), mitogen-activated protein kinase (MAPK), p70 ribosome protein S6 kinase (p70S6K), STAT protein 3 (STAT3), etc., and regulate the production and secretion of proinflammatory cytokines such as interleukin-1 (IL-1), IL-6, IL-15, IL-17, IL-23, and tumor necrosis factor-α (TNF-α); the anti-inflammatory cytokines include IL-4, IL-10, and IL-13, as well as the transforming growth factor-β (TGF-β) and interferon γ (IFN-γ), thereby improving UC symptoms [62] (Figure 3).

##### Regulation on the NF-κB-Signaling Pathway

The activation of the NF-κB pathway has been demonstrated to be closely related to intestinal inflammatory responses in both UC patients and animal models [63]. Under normal circumstances, NF-κB was combined with human nuclear factors κB inhibitory protein α (IκBα), which is present in an inactive state in the cytoplasm. The IκBα could be phosphorylated by I-κB kinase and then be degraded by the protease. Thereafter, free NF-κB was dissociated from the complex and then translocated into the nucleus to activate related target genes, ultimately leading to the overexpression of a variety of proinflammatory cytokines, such as cyclooxygenase (COX-2), inducible nitric oxide synthase (iNOS), IL-6 and TNF-α, which promoted the occurrence and development of intestinal inflammation [64,65]. DNBS was used to induce UC in rats and studied the effect of PJ on UC [31]. It was found that PJ could significantly inhibit the DNBS-induced release of NF-κB, IL-6, IL-1β, and TNF-α at the mRNA level, thus alleviating the damage of inflammatory cell infiltration to the intestinal mucosa. As an important active ingredient in the pomegranate peel, EA was supposed to show the ability to downregulate the score of *p*-IκBα/IκBα in the colon tissue of DSS-induced UC mice and reduce the expression of NF-κB, COX-2, iNOS, and other inflammatory mediators. As a result, the inflammatory response of intestinal tissue could be alleviated by the intervention of EA [36]. In another study conducted by Rosillo, it was indicated that in a TNBS-induced UC rat model, PE, EA, and their complex could reduce the protein levels of NF-κB, decrease the production of COX-2 and iNOS, and alleviate the damage of inflammatory cell infiltration to the intestinal mucosa [37]. In addition, compared with PE, the complex of PE and EA, as well as EA, possessed better activity in downregulating the expression of NF-κB, suggesting that EA may be an important active ingredient in anti-UC.

##### Regulation on the p70S6K-Signaling Pathway

The activation of the mammalian target of rapamycin (mTOR)/p70S6K-signaling pathways plays an important role in regulating cell proliferation and inflammation. In an inflammatory state, mTOR is phosphorylated and promotes the expression of its downstream protein p70S6K. The activated p70S6K can further promote cell proliferation and differentiation, and the proliferating and differentiated cells are subsequently damaged by inflammatory factors at the lesion site, leading to the production of more inflammatory factors and forming a malignant cycle [66]. Meanwhile, inflammatory factors such as TNF-α and IL-1β released from the site of the colitis lesion will further stimulate the proliferation of intestinal smooth muscle cells and promote the synthesis and release of IL-1β and IL-6 by fibroblasts, then amplifying inflammatory response and leading to tissue fibrosis [67]. It was found that compared with the DSS-induced chronic UC model group, the intervention of pomegranate beverage (PB) reduced the expression of p70S6K and ribosome protein S6 (RPS6) in rat colon tissue, which is the downstream protein of the mTOR pathway, thereby downregulating the levels of TNF-α, IL-1β, and IL-6 as well as upregulating the release of IL-10 [38]. The above results implied that PB might inhibit the production of inflammatory factors, reduce the infiltration of inflammatory cells into colon tissue, inhibit the proliferation of fibroblasts, and have potential anti-UC effects by downregulating mTOR pathway-related proteins.

Then, miR-145 was indicated to be activated in inhibiting angiogenesis, reducing inflammation, and suppressing cell proliferation; it may be a potential anti-inflammatory target. Kim et al. also investigated the effect of miR-145 on UC [39]. Hypoxia-inducible factor-1α (HIF-1α) also plays an important role in the regulation of angiogenesis, cell proliferation, and inflammation [68]. According to the reports, miR-145 regulates the expression of HIF-1α by identifying the promoter region of p70S6K1 [69]. PB was reported to inhibit the expression of TNF-α, IL-1β, COX-2, and iNOS in the colonic tissue of chronic UC rats induced by DSS modeling. The mechanism was supposed to be related to its up-regulation of miR-145 and downregulation of the p70S6K1/HIF-1α axis pathway [39].

##### Regulation on the MAPK-Signaling Pathway

MAPK, highly expressed in UC patients and TNBS-induced UC mouse models, is closely related to the development of UC. P38, c-Jun amino-terminal kinase (JNK), and extracellular signal-regulated kinase (ERK) are its main subtypes [70]. It was confirmed that the upregulation of iNOS and COX-2 expression levels in the colon tissue of TNBS-induced chronic UC mice was associated with the activation of p38 MAPK, JNK, and ERK1/2 [37]. According to their experiments, when compared with the model group, PE, EA, and their complex exhibited significant ability in decreasing the expression level of *p*-JNK/JNK, *p*-p38/p38, and *p*-ERK1/2/ERK1/2, thereby reducing the release of iNOS and COX-2. The level of MAPK pathway-related genes in rat colon tissue was detected through a real-time quantitative reverse transcription polymerase chain reaction (qRT–PCR) [38]. The results showed that compared with the model group, EA could downregulate the expression of MAPK pathway-related genes such as MAPK1, MAP2K2, SFN, and CDC42. Furthermore, it was found that the feed containing 0.5% EA could significantly decrease the phosphorylation level of p38 MAPK in the colon tissue of DSS-induced chronic UC mice (56 days) [36].

##### Regulation on the STAT3-Signaling Pathway

STAT3 is an important transcription factor that plays an important regulatory role in physiological processes such as inflammation, metabolism, and proliferation in the body [71]. Some research results have shown that the IL-6/STAT3-signaling pathway plays an important role in the occurrence and development of colitis. It was found that EA could inhibit the release of IL-6 and reduce the expression of phosphorylated STAT3 in colon tissue of UC mice, thus alleviating the inflammatory state of intestinal tissue [36].

All the above results suggested that pomegranate and its ellagitannins can reduce the production of inflammatory mediators and play anti-UC effects by blocking inflammatory-signaling pathways such as NF-κB, MAPK, p70S6K, and STAT3.

In addition, Larrosa found that in normal rats, urolithin had the best anti-inflammatory activity [25]. However, in the disease location of DSS-induced colitis in rats, ellagitannins played an anti-inflammatory role, rather than urolithin. In combination with clinical and animal experiments, EA and punicalagin were supposed to be active ellagitannins that showed an anti-inflammatory role in pomegranate fruits, peels, and flowers.

### 3.3. Intestinal Barrier Improvement Effects and Mechanisms

#### 3.3.1. Promoting the Expression of Tight Junction Protein

The intestinal barrier, which forms a complex physical barrier to safeguard colon tissue, is composed of epithelial cells, matrix elements, and the mucus layer released by epithelial cells. Tight junction proteins have a role in controlling the permeability of the paracellular barrier by dynamically regulating the closure of gaps between adjacent intestinal epithelial cells [72]. ZO-1, occludin, and other proteins comprise tight junction proteins. Its decreased expression will cause an increase in intestinal permeability, which will compromise the function of the intestinal barrier and contribute to the development of UC [73]. In the research of how polyphenols affect cell permeability, Caco-2, which is comparable to intestinal epithelial cells in form and function and have been shown to be able to replicate the absorption of intestinal epithelial cells in vivo, is frequently used [74]. To explore the impact of pomegranate and its ellagitannins on intestinal permeability, Zhao et al. utilized lipopolysaccharide (LPS)-induced Caco-2 to generate an intestinal barrier inflammation model [40]. They discovered that LPS dramatically decreased ZO-1 expression as compared to the control group. The expression level of ZO-1 considerably increased following the intervention of pomegranate peel polyphenols (PPP) and punicalagin, suggesting that PPP and punicalagin could reverse the rise of LPS-induced cell permeability. Punicalagin was also found to lessen the intestinal barrier disruption caused by DSS-induced reduction in ZO-1 and occludin proteins in mouse colon tissue [29].

#### 3.3.2. Inhibiting Apoptosis of Intestinal Epithelial Cells

The apoptosis of intestinal epithelial cells has been shown to impair the intestinal barrier in the early stages of UC patients, and the quantity of apoptotic bodies is strongly connected with the severity of lesions in UC patients [75]. Punicalagin can upregulate the expression of B-cell lymphoma-2 (Bcl-2) in the colon tissue of UC mice and has an antiapoptosis role, according to the reference [29].

**Figure 4:**
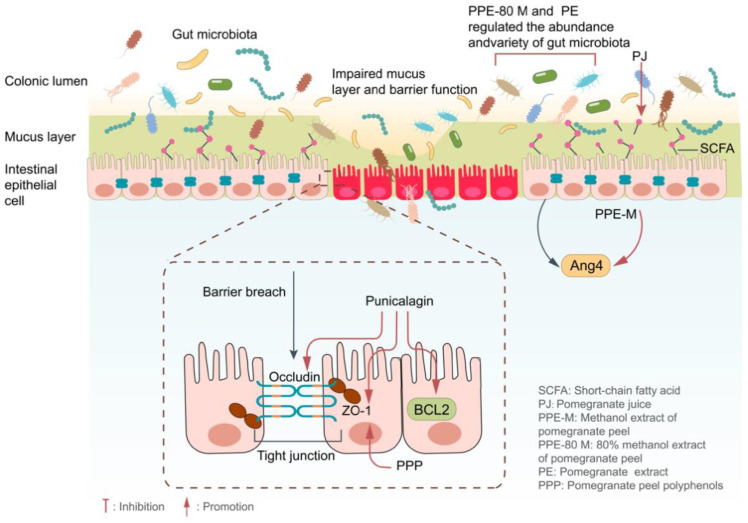
Pomegranate and its rich ellagitannin played an anti-UC role by improving the intestinal barrier and intestinal flora.

In conclusion, the primary pathogenic alteration in UC is the destruction of the intestinal mucosal barrier. By promoting the expression of tight junction protein and preventing intestinal epithelial cell death, pomegranate and its ellagitannins can aid in the restoration of the intestinal barrier (Figure 4).

### 3.4. Regulation of Gut Microbiota

The microflora of the human intestinal system is composed of 100-trillion distinct microorganisms, including bacteria, viruses, fungi, and protozoa [76]. The most common microorganisms are symbiotic bacteria that are beneficial to the body, helping to digest nutrients, preventing pathogen invasion, and maintaining normal intestinal function [77]. UC may be facilitated by bacterial invasion of the mucosa, according to the reference [78], who discovered mucosal bacteria in 83% of colon tissues from UC patients. In a DSS-induced UC mouse model, after consuming water containing 3% DSS for 12 h, bacteria were discovered in the mucus layer as well as on the surface and inside intestinal epithelial cells, while the mucus layer in the intestine was destroyed. This indicated that DSS may change the nature of the mucus layer, allowing bacteria to penetrate the mucus layer and then destroy intestinal epithelial cells. Before inflammatory cells infiltrated intestinal tissue and intestinal epithelial cells changed, bacterial invasion had already occurred, which suggested that bacterial invasion may be a precursor to the development of UC [79].

Short-chain fatty acids are metabolites of beneficial bacteria in the intestinal cavity, which are of great significance in promoting intestinal health and maintaining homeostasis [80]. PJ was reported to possess the ability to restore the reduction of short-chain fatty acids caused by DSS modeling and play an anti-UC role [41].

*Citrobacillus muris* (Cr) is a type of *Escherichia coli* that can naturally infect mice and cause diseases similar to human intestinal pathogenic bacterial infections. Except in highly sensitive mouse strains, the growth of Cr is limited to the colon in most of the mice, and it is an effective model for studying infectious colitis [81]. Angiopoietin-4 (Ang4) is a polypeptide protein that is primarily secreted by intestinal paneth cells and goblet cells. It can destroy intestinal pathogens with high specificity while leaving beneficial bacteria alone, thus preserving the balance of the gut microbiota [82]. It was found that the methanol extract of pomegranate peels (PPE-M) limited the spread of pathogenic bacteria Cr throughout the body and reduced the degree of damage to the colon caused by Cr by increasing the expression of Ang4 [42].

*Bacteroides* can boost the intestinal epithelial barrier function by promoting the growth of cells in the colon epithelium and the release of cytokines, which is widely seen as being advantageous to the body and can lessen the damage caused by enteropathogenic bacteria Cr infection to the intestinal mucosa [83]. According to George’s research, 80% methanol extract of a pomegranate peel (PPE-80 M) considerably enhanced the abundance of *Bacteroides* and decreased the abundance of *Firmicutes* in the intestines of mice with Cr infection [43]. It was found that compared to the model group, PE and urolithin increased the abundance of beneficial bacteria such as *Lactobacillus* and *Bifidobacterium* in the intestine of rats [25].

IL-10 knockout mice spontaneously form UC at 8–12 weeks [84]. It was suggested that PE increased the abundance of *Akkermansia muciniphila,* which was negatively correlated with the histological score of colitis in the intestinal tract of IL-10 knockout mice and decreased the abundance of *Paeniclostridium* and *Clostridium_sensu_stricto_1*, which were positively correlated with the histological score of colitis [35]. The above results showed that PPE could regulate the composition and abundance of gut microbiota and alleviate the degree of colitis injury in IL-10 knockout mice.

Gut microbiota disorder is one of the important characteristics of UC. Food intake rich in ellagitannins was proven to reduce the impact of intestinal pathogenic bacteria on the intestinal mucosa and increase beneficial bacteria in the intestinal tract, thus maintaining the balance of gut microbiota (Figure 4). Therefore, adjusting the composition and abundance of gut microbiota by consuming a diet rich in ellagitannins will become one of the research hotspots in the prevention and treatment of intestinal inflammation.

## 4. Discussion and Conclusions

The incidence rate of UC is the highest in Europe and the United States. However, in recent years, the incidence rate of UC in Asia and low-income countries has risen sharply [3]. UC is refractory and persistent, and one of its worst side effects—its propensity to progress to colorectal cancer—imposes a significant financial burden on patients and the healthcare system [85]. According to the estimation of the World Health Organization, about 80% of patients meet their basic health needs by using traditional medicine such as Chinese herbal extracts and their effective biological ingredients [86].

To protect gastrointestinal tissue, the hydroxyl group or other functional groups of tannins can precipitate a protective film of the tannin–protein complex locally, help prevent damage from mechanical or chemical stimulation, and encourage mucosal regeneration [6].

Pomegranate is widely used in traditional Chinese medicine and ethnic medicine such as Uyghur medicine, Tibetan medicine, and Mongolian medicine, and it has certain economic and medicinal value. Its fruits, flowers, and peels are rich in ellagitannins, which possess anti-inflammatory properties, antioxidants, and gut microbiota-regulating effects [25,28]. In this paper, the anti-UC effects and mechanisms of pomegranate’s different parts and their rich ellagitannins, especially the main components punicalagin and ellagic acid, reported in the literature in the last 20 years were reviewed. It was found that they mainly inhibit primary intestinal inflammation by regulating the key proteins in various inflammatory pathways such as NF-κB, MAPK, and p70S6k. Meanwhile, they can also relieve the symptoms of UC by reducing the level of oxidative stress, maintaining the integrity of intestinal epithelial cells and the diversity of gut microbiota (Table 1).

Ellagitannins are widely distributed in nature. Along with pomegranates, other fruits rich in ellagitannins, which are important natural products for the prevention and treatment of UC, include raspberries, blueberries, blackberries, cranberries, strawberries, walnuts, pistachios, hazelnuts, chebula, nuts, and Chinese herbal medicines [87,88]. Researchers are now interested in using ellagitannin-rich diets or plants in the field of functional foods. This also raises the possibility that UC can be prevented and controlled by dietary management.

It is difficult for ellagitannins to be absorbed in the stomach and small intestine. Although its important active ingredient, ellagic acid, has been proven to have good potential in treating UC in animal models, its wide application is limited due to its poor solubility, short half life, and other problems. Research has shown that the anti-UC impact of ellagic acid microsphere is noticeably superior to that of ellagic acid in prototype treatment in the rat UC model induced by DSS [30]. Therefore, greater consideration and in-depth study should be given to the chemical modification and transformation of ellagic acid to create a stable drug delivery system, which can increase its solubility and enhance its ability to target the colon.

Furthermore, most ellagitannin-rich dietary research currently focuses on the extract level, and the active ingredients have not been clearly elucidated. Scientific studies on the mechanisms of ellagitannins on human health have mostly been conducted at the animal or cellular level, and their effectiveness and safety in UC patients still need to be proven clinically.

**Figure 5:**
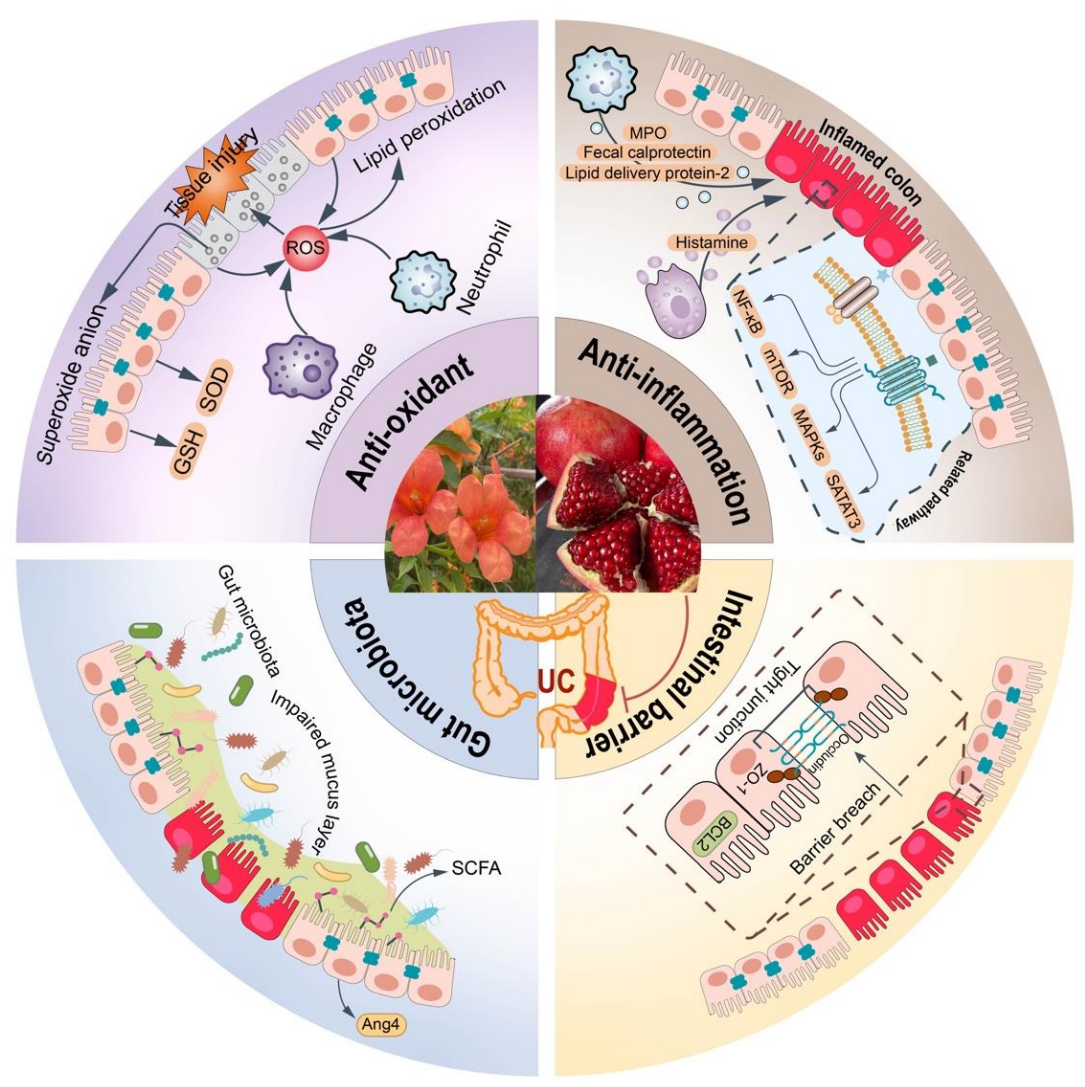
The anti-ulcerative colitis effect and mechanism of pomegranate and its rich ellagitannins.

In conclusion, in this paper, the anti-UC effects and mechanisms of pomegranate’s different parts and their rich ellagitannins, especially the main components punicalagin and EA, reported in the literature in the last 20 years were reviewed. It was found that they mainly inhibited primary intestinal inflammation by regulating the key proteins in various inflammatory pathways such as NF-κB, MAPK, p70S6K, and STAT3. Meanwhile, they can also relieve the symptoms of UC by reducing the level of oxidative stress, as well as maintaining the integrity of intestinal epithelial cells and the diversity of gut microbiota (Table 1, Figure 5). Greater consideration and in-depth study should be applied to the chemical modification and transformation of EA to create a stable drug delivery system, which can increase its solubility and enhance its ability to target the colon. Furthermore, it is commonly recognized that a variety of gut bacteria significantly contribute to the pathogenic process and immunity of IBDs, which include Crohn’s disease (CD) and UC. A thorough analysis of the antibody epitope repertoire specific to IBD revealed that 373 antibody responses were differentially prevalent in IBD patients as compared to normal patients. Of these, 17% were shared by both IBDs, 55% were specific to CD, and 28% were exclusive to UC. Among them, antibody reactivities against bacterial flagellins dominated in CD and were related to ileal involvement, fibrostenotic illness, and anti-Saccharomyces cerevisiae antibody (ASCA) positive. Although there was no discernible anti-agellin antibody response in UC patients, there was an overrepresentation of antibody responses against fibronectin-binding proteins, including fibronectin-binding Proteins A and B of *Staphylococcus aureus* and fibronectin-binding proteins SfbII and A of *Streptococcus pyogenes*, as well as *Parabacteroides johnsonii*, *Escherichia coli*, and *Bacteroides dorei* [89]. It is worth mentioning that ASCA and the anti-neutrophil cytoplasmic antibody (ANCA) are well-known serological markers for CD and UC, respectively, and their presence may predict the development of IBD [90]. However, there is a lack of related research on pomegranate and its ellagitannins. Related research urgently needs to be conducted for such an adjunctive dietary resource for celiac disease patients under a gluten-free diet.

## Figures and Tables

**Table 1:** Summary of the effect of pomegranate and its rich ellagitannins in anti-colitis.

Activity	Source	Model	Effect	Ref.
Antioxidation	Pomegranate juice (PJ)	–	↓ABTS, ↓DPPH, ↓DMPD, ↓FRAP	[28]
	Punicalagin	Mice/DSS-induced colitis	↓ROS	[29]
	Pomegranate flower hydroalcohol extract (PFHE), ellagic acid-rich fraction of pomegranate flower (EAOPF)	Mice/DSS-induced colitis	↓Superoxide anion level	[17]
	PFHE, EAOPF	Mice/DSS-induced colitis	↓TBARS	[17]
	Pomegranate extract (PE)	Rats/DSS-induced colitis	↓TBARS	[25]
	EA	Rats/DSS-induced colitis	↓TBARS	[30]
	PJ, punicalagin	Rats/DNBS-induced colitis	↓MDA	[31]
	Pomegranate peel extract (PPE)	Mice	↓MDA	[32]
	Punicalagin	Mice/DSS-induced colitis	↓MDA	[29]
	PPE	Mice	↑SOD1, ↑SOD2	[32]
	PJ, punicalagin	Rats/DNBS-induced colitis	↑SOD	[31]
	PJ	Rats/TNBS-induced colitis	↑GSH	[33]
Antiinflammation	PJ	IBD patients	↓FC	[34]
	PE	Mice/IL-10 deficient colitis model	↓LCN2	[35]
	PJ	Rats/TNBS-induced colitis	↓MPO	[33]
	PJ, punicalagin	Rats/DNBS-induced colitis	↓MPO	[31]
	EA	Mice/DSS-induced colitis	↓MPO	[36]
	PE, EA	Rats/TNBS-induced colitis	↓MPO	[37]
	PFHE, EAOPF	Mice/DSS-induced colitis	↓MPO	[17]
	PFHE, EAOPF	Mice/DSS-induced colitis	↓Histamine	[17]
	PJ	Rats/DNBS-induced colitis	↓NF-κB, ↓IL-6, ↓IL-β, ↓TNF-α	[31]
	EA	Mice/DSS-induced colitis	↓*p*-IκBα/IκBα, ↓NF-κB, ↓COX-2, ↓iNOS	[36]
	PE, EA	Rats/TNBS-induced colitis	↓NF-κB, ↓COX-2, ↓iNOS	[37]
	Pomegranate beverage (PB)	Rats/DSS-induced colitis	↓p70S6K, ↓RPS6, ↓TNF-α, ↓IL-1β, ↓IL-6	[38]
	PB	Rats/DSS-induced colitis	↑miR-145, ↓p70S6K1, ↓HIF-α, ↓TNF-α, ↓IL-1β, ↓COX-2, ↓iNOS	[39]
	PE, EA	Rats/TNBS-induced colitis	↓*p*-JNK/JNK, ↓*p*-p38/p38, ↓*p*-ERK1/2/ERK1/2, ↓COX-2, ↓iNOS	[37]
	PB	Rats/DSS-induced colitis	↓MAPK1, ↓MAP2K2, ↓SFN, ↓CDC42	[38]
	EA	Mice/DSS-induced colitis	↓p38 MAPK	[36]
	EA	Mice/DSS-induced colitis	↓*p*-STAT3, ↓IL-6	[36]
Improvement of intestinal barrier	pomegranate peel polyphenols (PPP), punicalagin	LPS-induced Caco-2	↑ZO-1	[40]
	Punicalagin	Mice/DSS-induced colitis	↑ZO-1, ↑Occludin, ↑Bcl-2	[29]
Regulation of intestinal microbiota	PJ	Mice/DSS-induced colitis	↑Short chain fatty acid	[41]
	methanol extract of pomegranate peel (PPE-M)	Mice/Cr-induced colitis	↑Ang4	[42]
	80% methanol extract of pomegranate peel (PPE-80 M)	Mice/Cr-induced colitis	↑*Bacteroidetes*, ↓*Firmicutes*	[43]
	PE	Rats/DSS-induced colitis	↑*Lactobacilli*, ↑*Bifidobacterium*	[25]
	PE	Mice/IL-10 deficient colitis model	↑*Akkermansia,* ↓*Paeniclostridium,* ↓*Clostridium_sensu_stricto_1*	[35]

Ref.: reference; ↑: up-regulated; ↓: down-regulated.
